# Deep learning-based tool affects reproducibility of pes planus radiographic assessment

**DOI:** 10.1038/s41598-022-16995-6

**Published:** 2022-07-28

**Authors:** Jalim Koo, Sangchul Hwang, Seung Hwan Han, Junho Lee, Hye Sun Lee, Goeun Park, Hyeongmin Kim, Jiae Choi, Sungjun Kim

**Affiliations:** 1 Department of Radiology, Gangnam Severance Hospital, Yonsei University College of Medicine, Seoul, Republic of Korea; 2 Research Institute of Radiological Science, Center for Clinical Imaging Data Science, Yonsei University College of Medicine, Seoul, Republic of Korea; 3 Department of Orthopedic Surgery, Gangnam Severance Hospital, Yonsei University College of Medicine, Seoul, Republic of Korea; 4 Department of Radiological Technology, Dongnam Health University, Suwon, South Korea; 5 Biostatistics Collaboration Unit, Yonsei University College of Medicine, Seoul, South Korea

**Keywords:** Musculoskeletal abnormalities, Translational research, Computer science, Medical imaging

## Abstract

Angle measurement methods for measuring pes planus may lose consistency by errors between observers. If the feature points for angle measurement can be provided in advance with the algorithm developed through the deep learning method, it is thought that the error between the observers can be reduced. A total of 300 weightbearing lateral radiographs were used for the development of the deep learning-based algorithm, and a total of 95 radiographs were collected for the clinical validation test set. Meary angle (MA) and calcaneal pitch (CP) were selected as measurement methods and measured twice by three less-experienced physicians with the algorithm-based tool and twice without. The intra- and inter-observer agreements of MA and CP measures were assessed via intra-class correlation coefficient. In addition, verification of the improvement of measurement performance by the algorithm was performed. Interobserver agreements for MA and CP measurements with algorithm were more improved than without algorithm. As for agreement with reference standard, combining the results of all readers, both MA and CP with algorithm were greater than those without algorithm. The deep learning algorithm tool is expected to improve the reproducibility of radiographic measurements for pes planus, especially by improving inter-observer agreement.

## Introduction

Pes planus (PP) is a common postural deformity in which the longitudinal arch of a foot collapses, which can be congenital or acquired^1^. Clinical manifestations vary from symptom absence to severe deformation according to the stage of disease^2^. PP diagnosis requires medical history, physical examination (such as inspection, range of motion and gait assessment, etc.), and radiographic evaluation, among which radiographic examinations are useful for assessing concurrent abnormalities, quantifying the degree of deformity, and determining the stage of the disease^3^. With respect to radiographic examination, reproducibility and objectiveness are highly required because radiographic examination is used not only for diagnosis, but also for surgical outcome measurement or postoperative evaluation of disease^4^.

There are various radiographic measurements to diagnose PP. Talo–first metatarsus angle (Meary angle), calcaneal pitch angle, medial cuneiform to fifth metatarsus height, and talar head uncoverage angle have been investigated and used as effective radiographic measurements^5,6^. Among them, calcaneal pitch and Meary angles have been reported to be more effective for assessing PP with respect to higher intraobserver and interobserver correlation coefficients compared with other radiographic assessment parameters^7,8^. However, there is no consensus method for defining the axis of each bone (talus, first metatarsus, calcaneus) used to measure these angles, and varied methods are suggested in the literatures to define them. Also, when the angles are manually measured, the diagnostic results may lack consistency as a result of human error and/or inexperience, which result in various intra-, interobserver variability for each measurement method^9^. This potential shortage of reproducibility could affect reliability of radiographic assessment. Therefore, for consistent and accurate radiographic measurement of PP, efforts are needed to increase the reproducibility of angle measurements.

Recently, deep learning based researches have been actively conducted to improve the accuracy, speed, and reproducibility of radiographic measurements and grading for musculoskeletal radiology field^10^. In this context, we speculated that deep learning-based algorithm might augment the reproducibility of radiographic measurements of PP by assisting objectivity of the measure. Hence, the purpose of this study was to assess whether deep learning-based algorithm augments reproducibility of radiographic measurements of Meary angle and calcaneal pitch to diagnose PP. Additionally, we assessed whether adding the algorithm in the measurement helps to increase the diagnostic accuracy of PP when being compared with the measurement without the algorithm.

## Results

### Study subjects

A total of 395 right and left weightbearing lateral radiographs from 395 adult (> 18 years) patients were included in our study (Supplementary Table 1). For algorithm reconstruction, 300 patients (mean age, 51.6 ± 17.1 years; range, 18–78; 114 males and 186 females) were selected. The test set for algorithm validation consisted of 95 radiographs from the 95 patients (mean age 54.8 ± 15.1 years; range, 18–82; 23 males and 72 females) were used for algorithm validation. The numbers of PP and non-PP images as reference standards re-defined by each radiologic criterion were 42 and 53 respectively by the Meary angle criteria, 64 and 31 respectively by the calcaneal pitch criteria.

### Preliminary algorithm assessment using the test set in algorithm development

#### Performance of bone boundary segmentation algorithm

The segmentation performance was measured by dice similarity coefficient, and the average measures were 96% (range, 0.845–0.981) for the talus, 93% (range, 0.803–0.964) for the first metatarsus and 98% (range, 0.948–0.988) for the calcaneus.

#### Agreement between human-measured and algorithm-measured angles

**Figure 1:**
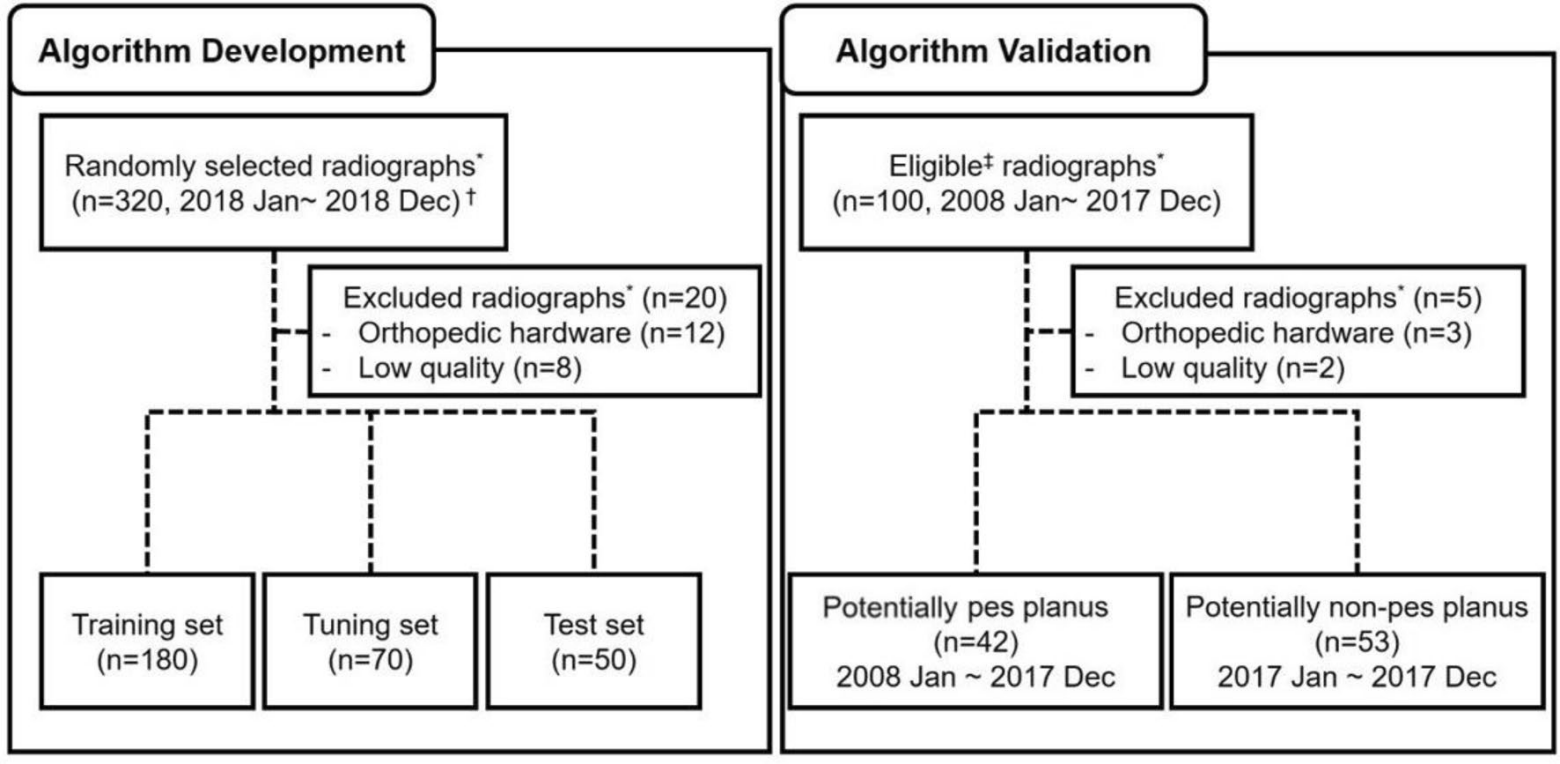
Flowchart of the study dataset assignment. *Weightbearing foot Lateral radiographs. ^†^Time period of image acquisition did not overlap among training, tuning, and test sets. ^‡^Radiographs from patients who were diagnosed as pes planus and from those who received treatment for reasons potentially other than pes planus through search for electronic medical records.

For preliminary validation of algorithm performance, Meary angle and calcaneal pitch were measured using the developed algorithm for the test set (randomly selected 50 patients, in Fig. 1), regardless of whether PP was diagnosed or not. When comparing the intraclass correlation coefficient (ICC) between the angle measured by the algorithm and by the human, the Meary angle was 0.893 (95% CI 0.819–0.938), and the calcaneal pitch was 0.992 (95% CI 0.986–0.996).

### Algorithm validation

#### Measurement reproducibility

As for intraobserver agreement, except for the calcaneal pitch measured by R3, ICC with algorithms for Meary angle and calcaneal pitch were greater than ICC without algorithms in all readers. However, among the comparisons, ICC for calcaneal pitch in R1 and those for Meary angle in R2 only showed a statistically significant increase (P < 0.05) respectively, with the use of algorithm compared to without the algorithm (Table 1).

**Table 1:** Intraobserver interclass correlation coefficient.

ICC (95%CI)	Without algorithm	With algorithm	Difference	P value
**Reader 1**				
Meary angle	0.991 (0.982–1.000)	0.993 (0.987–1.000)	0.003 (− 0.005 to 0.01)	0.4782
Calcaneal pitch	0.989 (0.979–1.000)	0.998 (0.996–1.000)	0.009 (< 0.001–0.019)	0.0002
**Reader 2**				
Meary angle	0.899 (0.814–1.000)	0.986 (0.973–1.000)	0.087 (0.007–0.166)	< 0.0001
Calcaneal pitch	0.989 (0.979–1.000)	0.992 (0.985–1.000)	0.003 (− 0.006 to 0.012)	0.5036
**Reader 3**				
Meary angle	0.957 (0.92–0.994)	0.968 (0.94–0.996)	0.011 (− 0.023 to 0.045)	0.5346
Calcaneal pitch	0.993 (0.986–0.999)	0.986 (0.974–0.998)	− 0.007 (− 0.017 to 0.004)	0.1791

As for interobserver agreement, ICC with algorithms for Meary angle and calcaneal pitch showed a statistically significant increase (P < 0.0001) compared to ICC without algorithm (Table 2).

**Table 2:** Interobserver interclass correlation coefficient.

ICC (95%CI)	Without algorithm	With algorithm	Difference	P value
Meary angle	0.814 (0.757–0.871)	0.955 (0.939–0.97)	0.141 (0.092–0.189)	< 0.0001
Calcaneal pitch	0.974 (0.965–0.983)	0.984 (0.978–0.989)	0.01 (0.003–0.016)	< 0.0001

#### Diagnostic performance

The results of comparing the agreement between the reference standard and the reader's measurement results are as follows. In the case of Meary angle, the agreement with the reference standard was statistically significantly increased in all readers when measured using the algorithm. Combining all the results from reader together as for ICC without algorithms for Meary angle was 0.815(95% CI 0.776–0.854) and those with algorithms was 0.896 28(95% CI 0.873–0.919) also showing statistically significant increase of ICC with algorithm use (P < 0.05). As for calcaneal pitch, ICC of R3 and that from combining all three readers were higher with algorithm than without algorithm with statistical significance, although no statistically significant increase of ICC with the use of algorithm were seen in R1 and R2 (Table 3).

**Table 3:** Agreement with reference standard.

	Meary angle				Calcaneal pitch			
	Without algorithm	With algorithm	Difference	p-value	Without algorithm	With algorithm	Difference	p-value
Reader1	0.812 (0.743–0.881)	0.872 (0.824–0.92)	0.06 (0–0.12)	0.0304	0.978 (0.969–0.987)	0.978 (0.969–0.987)	0 (− 0.009 to 0.009)	> 0.9999
Reader2	0.726 (0.631–0.821)	0.898 (0.859–0.937)	0.172 (0.09–0.254)	< 0.0001	0.969 (0.957–0.981)	0.967 (0.954–0.98)	− 0.002 (− 0.015 to 0.011)	0.7584
Reader3	0.838 (0.778–0.898)	0.913 (0.879–0.947)	0.075 (0.023–0.127)	0.001	0.943 (0.921–0.965)	0.979 (0.971–0.987)	0.036 (0.016–0.056)	< 0.0001
Reader1 + 2 + 3	0.815 (0.776–0.854)	0.896 (0.873–0.919)	0.081 (0.047–0.115)	< 0.0001	0.963 (0.955–0.971)	0.974 (0.968–0.98)	0.011 (0.003–0.019)	0.0024

There was no statistically significant increase in the sensitivity, specificity and accuracy of diagnosis according to the use of the algorithm in all cases except for the specificity of the Meary angle by R2 (Table 4).

**Table 4:** The sensitivity and specificity of Meary angle and calcaneal pitch.

	Meary angle			Calcaneal pitch		
	Without algorithm	With algorithm	Comparison p-value	Without algorithm	With algorithm	Comparison p-value
**Sensitivity**						
Reader1	100.00 (100.00–100.00)	95.24 (88.80–100.00)	0.1468	95.31 (90.13–100.00)	96.88 (92.61–100.00)	0.3041
Reader2	73.81 (60.51–87.11)	85.71 (75.13–96.30)	0.0538	100.00 (100.00–100.00)	96.88 (92.61–100.00)	0.1452
Reader3	97.62 (93.01–100.00)	90.48 (81.60–99.35)	0.1752	98.44 (95.40–100.00)	96.88 (92.61–100.00)	0.3128
Reader1 + 2 + 3	90.48 (85.35–95.60)	90.48 (85.35–95.60)	> 0.9999	97.92 (95.90–99.94)	96.88 (94.41–99.34)	0.3957
**Specificity**						
Reader1	77.36 (66.09–88.63)	84.91 (75.27–94.54)	0.0386	93.55 (84.90–100.00)	96.77 (90.55–100.00)	0.3259
Reader2	96.23 (91.10–100.00)	92.45 (85.34–99.56)	0.3241	93.55 (84.90–100.00)	96.77 (90.55–100.00)	0.2882
Reader3	83.02 (72.91–93.13)	92.45 (85.34–99.56)	0.0597	93.55 (84.90–100.00)	96.77 (90.55–100.00)	0.3259
Reader1 + 2 + 3	85.53 (80.07–91.00)	89.94 (85.26–94.61)	0.0892	93.55 (88.56–98.54)	96.77 (93.18–100.00)	0.176
**ACC**						
Reader1	87.37 (80.69–94.05)	89.47 (83.30–95.65)	0.3977	94.74 (90.25–99.23)	96.84 (93.33–100.00)	0.1617
Reader2	86.32 (79.40–93.23)	89.47 (83.30–95.65)	0.3731	97.89 (95.01–100.00)	96.84 (93.33–100.00)	0.5437
Reader3	89.47 (83.30–95.65)	91.58 (85.99–97.16)	0.5731	96.84 (93.33–100.00)	96.84 (93.33–100.00)	> 0.9999
Reader1 + 2 + 3	87.72 (83.91–91.53)	90.18 (86.72–93.63)	0.2534	96.49 (94.35–98.63)	96.84 (94.81–98.87)	0.757

## Discussion

Our study demonstrated that a deep learning-based tool enhanced the reproducibility of PP radiographic assessment.

Physicians use various methods to measure the Meary angle and calcaneal pitch according to their preference. In this study, we selected a method that our institution physicians have been using among those various methods. The method we chose was reported as one of the most reproducible methods in a previous comprehensive^9^.

For evaluation of measurement reproducibility, intraobserver and interobserver agreement was assessed by comparing between measurement 'with algorithm' and measurement 'without algorithm'. Increase of intraobserver agreement in algorithm-assisted measurement were seen only in Meary angle of R1 and calcaneal pitch of R2 with statistical significance. We speculated that unexpectedly marginal increase of intraobserver agreement contributed to the time interval between the measurement session. A week was presumably not long enough to completely forget the previous measurement circumstance. In contrast, the interobserver agreements for Meary angle and calcaneal pitch were higher with statistical significance when being measured with algorithm. Many studies have reported varied inter- and intraobserver agreement of the Meary angle and calcaneal pitch measurements^9^. As for Meary angle, the intraobserver ICC values were reported to be 0.71–0.96, and the interobserver ICC values were reported to be 0.59–0.86. As for calcaneal pitch, intraobserver ICC values were reported to be 0.68–0.98, and the interobserver ICC values were reported to be 0.76–0.98. Those results showed comparable or lower reproducibility than those of the current study, and it appears to be meaningful that our study showed that the use of algorithm can increase the reproducibility between the readers.

Another consideration related to ICC is the variation in dice similarity coefficient. Variation of dice similarity coefficient of talus and the first metatarsus was larger than that of calcaneus. This means that it is possible that the algorithm presented some features based on somewhat inaccurate segmentation to the physicians for only a certain subset of images. This may be why the ICC of Meary angle was still lower than that of the calcaneal pitch even when using the algorithm. However, in both the calcaneal pitch and the Meary angle, the ICC when using the algorithm was higher than the ICC when not using the algorithm. I believe that this means that the developed algorithm still has the effect of increasing the consistency between measurements of the Meary angle, even though there is a variation in the segmentation accuracy of talus and first metatarsus.

In order to evaluate whether the algorithm enhances diagnostic accuracy in the diagnosis of PP in addition to the reproducibility augmentation in our study, the following two approaches were used. First, in the agreement between the reference standard measurements created by two experienced physicians and the measurements of each less experienced reader, algorithm enhanced the concordance of the measured values with the reference standard except for the calcaneal pitch measurement for R1 and R2. We speculate that the relatively limited merit of calcaneal pitch is attributed to the fact that the feature point definition for the calcaneal pitch was relatively clearer than that of the Meary angle. Second, in the comparison of sensitivity, specificity, and accuracy between with and without algorithm, there was no statistically significant difference for each value unexpectedly. The agreement between the reference standard and the measured value itself tends to increase through the use of the algorithm, but the sensitivity, specificity, and accuracy of diagnosing PP do not increase statistically significantly. The cause of this unexpected discrepancy seems to be that the increase in diagnostic performance by use of the algorithm was limited possibly due to limited validation data volume, and this should be further verified using more data in actual clinical situations. However, we believe that this finding is significant in that it improved the reproducibility among less-experienced readers without compromising the diagnostic performance by using the algorithm.

A similar attempt like our study was made before. A group of investigators demonstrated semiautomatic angle measurements for PP diagnosis based on lateral radiographs to improve the reliability of measurements as did we in this study^11^. The investigators implemented automatic calculation of calcaneal-fifth metatarsus angle by using manually segmented calcaneus and fifth metatarsus, and they were not focused on the automation of the manual segmentation. As they stated in their report, segmentation is time-consuming and tedious work, and automation by manual segmentation cannot fully fulfill the goal of automation^11^. We developed automatic bone segmentation algorithm,a detecting outlines of the bones and the reference points for angle measurement using a deep learning algorithm. We believe that this is the first attempt to develop an automatic segmentation tool and resultant genuine automation of angle measurement for PP assessment.

Our study focused on physicians with little experience because we thought that reproducibility matters, particularly for the less experienced. Even in the absence of an algorithm, the role of the algorithm may not be significant to an experienced physician who already may have high reproducibility of measurements. In addition, it can be difficult for a less-experienced physician to manually measure angles proficiently with methods learned with just a few examples. This information bias could affect reproducibility and measurements with little clinical experience may result in low accuracy and reproducibility concurrently. We believe that improvement of the reproducibility by algorithm deployment tested by our study support that this potential disagreement of the measurement by the less experienced readers can be minimized through the implementation of the algorithm.

Our study has some limitations. First, because this study was designed as a retrospective study, controls are often recruited by convenience sampling, so they are not representative of the general population and are prone to selection bias. Also, the retrospective aspect may introduce information bias^12^. In order to minimize the above bias, the data set of the patients was randomly selected from the clinical records, and the each set was classified in the order of the test dates so as not to lose the randomness^13^. In addition, observers performed blind measurements without knowing whether or not the patient's PP was diagnosed^14^. Second, a total of 395 images of 395 patients were used, and the number of cases can be considered small for deep learning-based development. However, a study by Zheng et al^10^ that enrolled a total of 179 patients and analyzed the images showed convincing research results, so the number of 395 cases analyzed in this study is believed to be sufficient to show feasibility. Third, there could also be an issue that why the anteroposterior (AP) view was not included in the assessment^15,16^. The angle measured on the AP view has a limitation in showing the lateral column deformity compared to the angles measured on the lateral view. According to former investigation^17^, on the AP views, the calcaneal-fifth metatarsal angle addresses lateral column deformity but is more correlated to forefoot abduction. The AP calcaneal-first metatarsal angle is difficult to measure, and the AP talar-first metatarsal angle is more reliably used to assess abduction and adduction. Therefore, we conducted research focusing on the angles that can be measured on the weightbearing lateral view. However, in general, the AP view has great value in accurately locating the anatomical location of major deformities, so future studies on the AP view for the diagnosis of PP should be further evaluated. Fourth, in this study, only internal validation was performed with the data set of our institution, although we believe that this drawback has partly been mitigated by separating the test data period from the training data period. In future studies, external validation using data from different institutions would verify the robustness of our algorithm.

In conclusion, we demonstrated that the deep learning algorithm tool augmented the reproducibility of radiographic measurements for PP, particularly for the interobserver agreement, without loss of diagnostic performance. It is expected that the reliability of radiographic diagnosis will be increased by securing the reproducibility of the measurement for the diagnosis of PP.

## Materials and methods

This retrospective study was approved by the institutional review board of Yonsei University, Gangnam Severance Hospital where this study was conducted (IRB No 3-2020-0127). All methods were performed in accordance with the ethical standards of Helsinki Declaration. Because the data used in this retrospective study were fully de-identified to protect patient confidentiality, the requirement for informed consent was waived by the institutional review board of Yonsei University, Gangnam Severance Hospital.

### Study population and radiograph data

To increase the independence of the data to be used for verification of the developed algorithm, the data to be used for algorithm development and the ones to be used for algorithm validation were divided as in Fig. 1.

First, from the medical record, we obtained a list of adult patients aged 18 years or older who complained of foot abnormalities to the hospital and took weightbearing lateral radiographs from January 2018 to December 2018. Of these, 320 patients were randomly selected, and radiographs were obtained for use in algorithm development. For algorithm development, right or left foot radiographs from each patient were randomly selected, anonymized, and sent to computer storage via the Picture Archive and Communication System (PACS). Among the radiographs, 12 radiographs were excluded because there were orthopedic hardware, and eight radiographs were excluded due to low quality. Regardless of whether or not PP was diagnosed, the X-ray images were divided into training (n = 180, 60%), tuning (n = 70, 23%) and test (n = 50, 17%) sets. Image acquisition periods did not overlap between the training, tuning, and test sets.

To evaluate the performance of the developed algorithm to diagnose PP, data to be used for algorithm validation were obtained from two separate adult patient lists. One was from potentially PP patients and the other from potentially non-PP patients, respectively, as follows. The list of potentially PP patients diagnosed with PP and who underwent tendon transfer surgery from January 2008 to December 2017 was obtained (n = 42) through a search of medical records. Additionally, the list of potentially non-PP patients was obtained by searching through medical records for patients who underwent weightbearing lateral radiography when the primary diagnosis by the clinician was not PP from January 2017 to December 2017. Among them, radiographs of inadequate quality, 53 patients were randomly selected.

As described above, the data were obtained by dividing the data into two groups of PP and non-PP intended to include PP images sufficiently. The reason was that the possibility of not achieving generalization of the algorithm could not be excluded as the data images were randomly selected regardless of the presence or absence of PP during its development process.

### Angle measurement for diagnosis of pes planus

Meary angle and calcaneal pitch were adopted to assess PP in this study because, as stated above, they have been proposed as being more effective compared with other measurements^7,8^.

**Figure 2:**
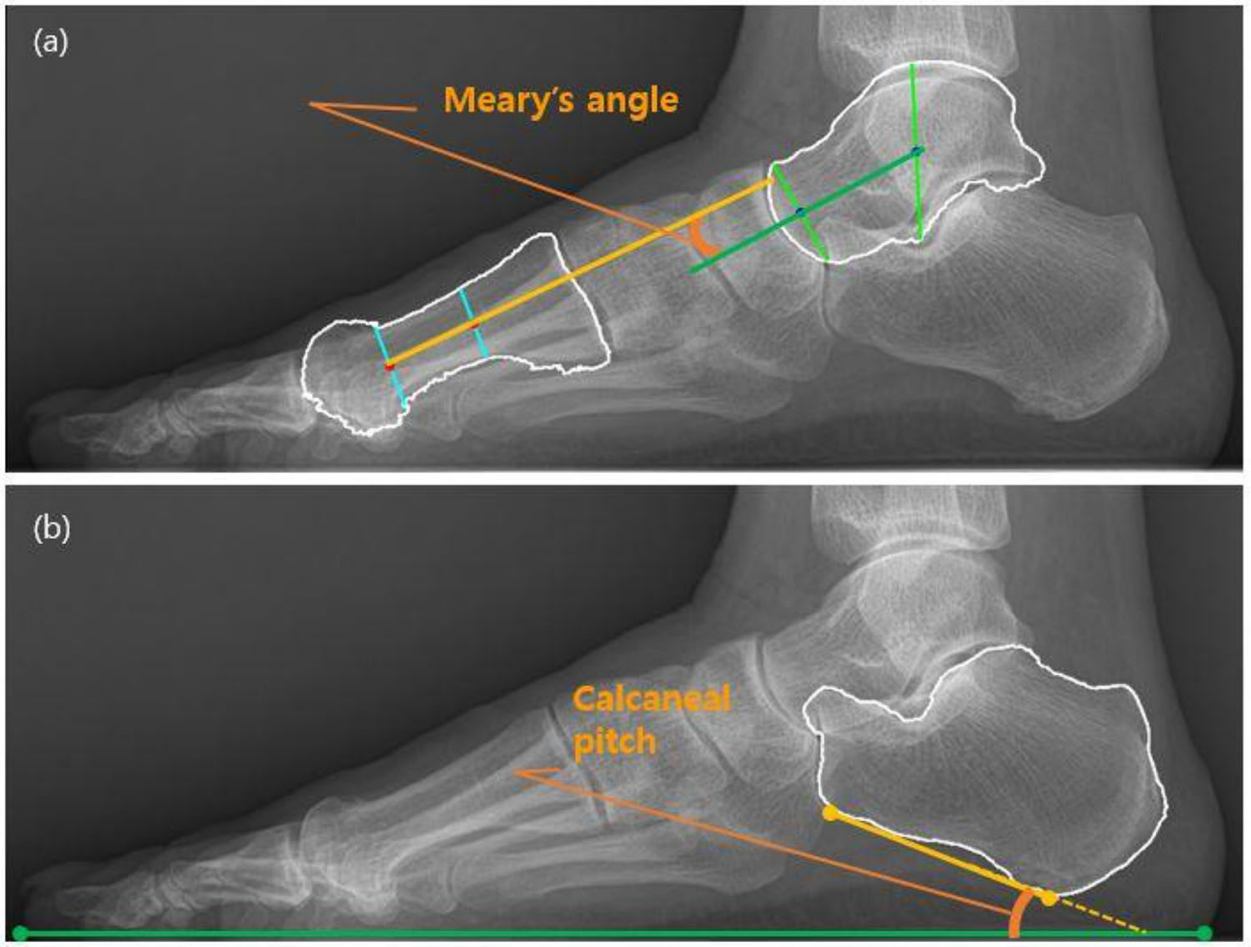
A weightbearing lateral radiograph of a normal foot of a 53 year old female, showing measurement of the Meary angle and calcaneal pitch**.** (**a**) Meary angle is the angle between the long axis of the talus and the long axis of the first metatarsus. First, the longitudinal axis of the talus is a line drawn through two mid-points. (Green line) One is a mid-point between the cephalad and caudad margins of the talar body, (Longer light green line) and the other is a mid-point between the cephalad and caudad margins of talar neck. (Shorter light green line) The metatarsus axis was determined using the following method. The long axis of the first metatarsus (Yellow line) is drawn by connecting two mid-points equidistant from the cephalad and caudad margins of the first metatarsus at the proximal and distal metaphysial diaphysial junction (blue lines). (**b**) Calcaneal pitch is the angle formed between the line outlining the inferior border of the calcaneus (yellow line) and the weight bearing surface (green line).

Meary angle is the angle between the long axis of the talus and the first metatarsus. There are many methods to line drawing to represent the long axis of the talus and first metatarsus, but we adopted the method illustrated in Fig. 2a^16,17^. Meary angle is considered normal within the range of − 4° to 4°, where the angle is defined as positive when the axis of the metatarsus is plantarly tilted less than that of the talus. An angle is less than − 4° is considered PP, and this criterion was adopted as the cut-off value for PP diagnosis^2^.

Calcaneal pitch is the angle formed between the line outlining the inferior border of the calcaneus and the weightbearing surface^18,19^. Generally, the range of calcaneal pitch from 18° to 20° is considered normal and decreased calcaneal pitch is considered PP^2^. In this study, the angle that is less than 18° was adopted as the cut-off value for PP (Fig. 2b).

### Development of automatic algorithm and measurement tool

Technical details on the development of automatic and manual measurement tools for Meary angle and calcaneal pitch are covered in the Supplementary. To briefly summarize the contents mentioned in the Supplementary, an algorithm for segmenting the talus, first metatarsus, and calcaneus was developed using the SegNet Model^20^. The algorithm was further developed to geometrically detect landmarks used for angle measurement using the boundaries of the three bones segmented by the algorithm. In order to achieve the purpose of this study to compare the use and non-use of the automatic measurement algorithm, two tools were developed to measure the Meary angle and the calcaneal pitch. One was a tool with the automatic measurement algorithm stated above, and the other was a tool without an algorithm. A tool equipped with an automatic measurement algorithm was developed so that the measurer can adjust the landmark suggested by the algorithm if it is judged inappropriate by the measurer.

### Algorithm validation by physicians

Using the test set for clinical validation, Meary angle and calcaneal pitch were independently measured by three less-experienced physicians (R1, R2, and R3) with and without an algorithm-based tool. R1 and R2 were first-year fellow trainees as musculoskeletal radiologists. R3 was a third-year radiology resident trainee. The readers were less experienced radiologists who did not participate in program development. They learned how to measure the Meary angle and calcaneal pitch and were trained on how to use the two programs with/without algorithm. Each reader measured angels of 10 patients not used in this study under the supervision of the experienced radiologist (S.K.).

For measuring intraobserver interclass correlation coefficient respectively, ten unilateral weightbearing images from the potentially PP group and the potentially non-PP group, a total of 20 images, were selected randomly. Therefore, Meary angle and calcaneal pitch were measured once more by three less-experienced physicians. The measurements were done with intervals between each measurement session being 1-week for each reader.

### Statistical analysis

With reference to the landmark displayed along with manual segmentation, the degree of agreement between the reference angle value and the value measured by the algorithm was obtained using ICC, and the diagnostic performance of the algorithm was evaluated.

For assessment of reproducibility for Meary angle and calcaneal pitch measurement, intraobserver and interobserver agreement were verified through single measures ICC by using one-way and two-way random models, respectively. To evaluate whether there is a difference in the degree of agreement between the reference standard value and the reader's measurement value without and with the algorithm, the single measures ICCs calculated using the two-way random model.

A difference between ICCs of without and with algorithm and 95% confidence interval (CI) for the difference were obtained^21^. When comparing ICCs of without and with algorithm for each reader, we took into account the correlation among the values on the same subject. When comparing the ICC by combining the results of the three readers(R1 + R2 + R3), since it was measured on the same subjects using without algorithm or with algorithm, the correlation within the subject was considered. However, the correlation between readers was not considered due to the limitations of the formula. Based on the report by Altman, an ICC of 0.81–1 was considered very good, 0.61–0.8 good, and 0.41–0.6 moderate^22^.

The reference standard was set to result measured by the experienced radiologist manually using the 'without algorithm tool'. The reference standard for diagnosis of PP in all patients was determined only by radiographic analysis using the measured angle excluding clinical information, wherein the reference standard was made for Meary angle and calcaneal pitch, respectively. Based on reference standard, the ‘potentially PP patient group’ clinically diagnosed by orthopedic physicians was re-diagnosed by only radiographic evaluation using Meary angle and calcaneal pitch, respectively, and the patient and non-patient groups were divided accordingly. In the same way, the ‘potentially non-PP patients’, the people who underwent weightbearing lateral radiographs, although the clinically primary diagnosis is not PP, from 2016 to 2017, were diagnosed only by angle and divided into the patient and non-patient groups (Table 5).

**Table 5:** Summary of reference standard based on radiologic criteria and electronic medical record.

	Number of radiographs based on EMR^c^	Meary angle		Calcaneal pitch	
		Pes planus	Non-pes planus	Pes planus	Non-pes planus
Potentially pes planus^a^	42	31	11	41	1
Potentially non-pes planus^a^	53	11	42	23	30
The number of radiographs per reference standard based on radiologic criteria^b^		42	53	64	31

^a^Diagnosis based on record or electronic medical record.

^b^Diagnosis based on radiologic criteria of Meary angle and calcaneal pitch respectively.

^c^Electronic medical record.

We compared the sensitivity, specificity, and accuracy of each reader between without and with algorithm measurements for Meary angle and calcaneal pitch, respectively. P-value was obtained using the bootstrap method (1000 replications).

## Supplementary Information

Supplementary Information.

## Data Availability

According to the data policy of Yonsei University Medical Center, where this research was conducted, permission from the “Data Asset Review Committee” and the “Data Review Board” must be obtained in order to export or disclose data. Therefore, it is necessary to pass the administrative procedures of Yonsei Medical Center in order to provide it to a desired external researcher or external institution. This approval process is exempted for research conducted by internal researchers, and therefore was not needed to obtain for the current research. Therefore, we cannot provide data at this point in time, but upon request, it can be provided after the completion of the processes stated above.
