# Author Correction: Deep learning-based tool affects reproducibility of pes planus radiographic assessment

**DOI:** 10.1038/s41598-022-19434-8

**Published:** 2022-09-07

**Authors:** Jalim Koo, Sangchul Hwang, Seung Hwan Han, Junho Lee, Hye Sun Lee, Goeun Park, Hyeongmin Kim, Jiae Choi, Sungjun Kim

**Affiliations:** 1 Department of Radiology, Gangnam Severance Hospital, Yonsei University College of Medicine, Seoul, Republic of Korea; 2 Research Institute of Radiological Science, Center for Clinical Imaging Data Science, Yonsei University College of Medicine, Seoul, Republic of Korea; 3 Department of Orthopedic Surgery, Gangnam Severance Hospital, Yonsei University College of Medicine, Seoul, Republic of Korea; 4 Department of Radiological Technology, Dongnam Health University, Suwon, South Korea; 5 Biostatistics Collaboration Unit, Yonsei University College of Medicine, Seoul, South Korea

Correction to: *Scientific Reports* 10.1038/s41598-022-16995-6, published online 28 July 2022

In the original version of this Article Jalim Koo and Sangchul Hwang were omitted as equally contributing authors.

The original Article has been corrected.

