# Supplementary material for: Deep learning-based tool affects reproducibility of pes planus radiographic assessment
Source: Sci Rep. 2022 Jul 28;12:12891. doi: 10.1038/s41598-022-16995-6 (PMC9334287; doi:10.1038/s41598-022-16995-6)
Supplement: Supplementary file 1 — Supplementary Information. [file 41598_2022_16995_MOESM1_ESM.pdf]

Supplementary information

Deep learning-based tool affects reproducibility of pes planus radiographic assessment

Jalim Koo, Sangchul Hwang, Seung Hwan Han, Junho Lee, Hye Sun Lee, Goeun Park, Hyeongmin Kim, Jiae Choi, Sungjun Kim.

Supplementary Note

Development of automatic tool for angle measurement

Automatic measurement tool for Meary angle and calcaneal pitch was planned to be based on segmentation of the corresponding bones and the point definition through geometric point detection based on the segmented boundary (Supplementary Fig. S1).

Segmentation algorithm for osseous structures

To feed the images for training, the masks of talus, first metatarsus and calcaneus were manually segmented using Adobe Photoshop CC 2018 software (Adobe Systems Inc., San Jose, CA, USA) and served as "ground truth". The masks were labeled by a radiology technician (J.L) under the guidance of an experienced radiologist (S.K.). Two separate labels were created for each bone, one for the bone location label and the other for the bone boundary label. The bone location labels were created by drawing boxes so that the center of each bone is located in the center including entire portion of the target bone. The bone boundary label was created by drawing along the boundary of each bone.

Segmentation algorithm was developed into two steps based on the two sets of ground truths. The first step was to find the region of interest where the target bone is located, and the second step was to segment the boundaries of the target bones in the image region of interest. SegNet model[1] was used in both steps (Supplementary Fig. S2). In the first step, the SegNet model was trained by using the bone location label (talus, the first metatarsus, and calcaneus) and implemented with MATLAB R2018b on a GeForce GTX 1080Ti graphics processing unit. Parameters used for the training was like follows: batch size of 4; maximum iteration number of 120 echoes; and learning rate of 0.01. The images were resized to 497 X 351 with pixel intensities scaled into 0 to 1 for training. In the second step, the SegNet model was trained with batch size of 4, maximum iteration number of 120 echoes, and learning rate of 0.01. The region of interest of the target bones used for training was cropped from the original image in 600 X 600 pixels for talus and the first metatarsus and in 600 X 300 pixels for calcaneus with intensities scaled into 0 to 1 and were fed to the training model with bone boundary label.

For preliminary testing of segmentation, 50 radiographs tagged as a test set were segmented automatically using the trained algorithm and the results were compared with the results of manual segmentation by humans.

To evaluate the performance of segmentation algorithm, dice similarity coefficient (DSC) was implemented which uses the automated segmentation mask and the human-annotated segmentation mask for the similarity comparison based on the following equation.

$$\mathrm{DSC} = \frac{2|A \cap B|}{|A| + |B|}$$

In this equation, │A│ is the sets of foreground pixels in the human annotation and │B│is the corresponding sets of foreground pixels in the automated segmentation result. │A∩B│is the intersection of the two sets. DSC greater than or equal to 0.7 have been considered as excellent agreement between two segmented regions in previous investigations,3,28 so, we decided to use the segmentation algorithm when the DSC value is greater than 0.7 in the assessment of test set.

Prediction of landmark used for angle measurement

The landmarks for Meary angle and calcaneal pitch were labeled by an experienced radiologist and an orthopedic surgeon (17 years of experience in foot radiography assessment) in consensus using the method stated above to obtain reference standard of the angles.

For measurement of Meary angle, we used a registration algorithm called 'ICP (iterative closest point)' with the reference standard segmentation contour made as being stated above for talus and the first metatarsus. ICP is an algorithm used in the process of accurate registration of point cloud data. Given two clouds of points (a reference and a source), the algorithm finds correspondences between the point clouds and tries to determine the translation and rotation matrices[2]. First, the binary image generated from the SegNet model and the mask image used for training were matched through an ICP method and similarity was calculated. Then, 10 images with high similarity were extracted from the training data. Next, as shown in Supplementary Fig. S3, the distance between the landmarks included in the 10 extracted images and the contour of the generated binary image was calculated, and positions of points with the closest distance were selected as landmarks of the generated binary image. Then, the midpoint of the landmarks was extracted and the centroid of the 10 extracted midpoints was detected to form a mid-axis of talus and 1st metatarsus.

For measurement the calcaneal pitch, the convex hull corresponding to the binary image was calculated. The convex hull is the smallest set of convex that contains a given point or region as a set. the calcaneal pitch was measured by calculating the lowest point and the inflection point after dividing the convex set into clusters by performing k-means clustering based on the calculated convex set as shown in Supplementary Fig. S4.

Measurement Tool Development

To create a PP radiographic evaluation tool, we used GUIDE (Graphical User Interface Development Environment) as a tool for creating a graphical user interface in MATLAB. We developed two measurement tools. One was a tool equipped with an automatic

measurement algorithm, and the other was one without the algorithm. The tool equipped with the developed automatic measurement algorithm (with-algorithm tool) was developed to automatically measure the angles stated above so that the algorithm finds and shows landmarks used for angle measurement, and if these landmarks are inappropriate, the physician can adjust the landmarks (Supplementary Fig. S5). The tool unequipped without the algorithm (without-algorithm tool) was developed to allow the physician to designate landmarks used for angle measurement without the aid of the algorithm, and to calculate the angle using these points (Supplementary Fig. S6).

Supplementary References

1. Badrinarayanan, V., Kendall, A. & Cipolla, R. SegNet: A Deep Convolutional Encoder-Decoder Architecture for Image Segmentation. *IEEE Trans Pattern Anal Mach Intell* **39**, 2481-2495. https://doi.org/10.1109/tpami.2016.2644615 (2017).
2. Chen, Y. & Medioni, G. Object modelling by registration of multiple range images. *Image and Vision Computing* **10**, 145-155. https://doi.org/https://doi.org/10.1016/0262-8856(92)90066-C (1992).

Supplementary Table

Supplementary Table 1 Summary of Demographic Information for Data set

**Demographic parameters**	**Data for algorithm reconstruction**				**Test set**		
	Training	Tuning	Test	**total**	Pes planus	Potential non pes planus	**total**
**No. of patients**	180	70	50	300	42	53	95
**Mean age(y) ***	51.6±17.4 (18-78)	53.2±16.8 (20-77)	49.9±17.7 (18-75)	51.6±17.1 (18-78)	63.5±12.6 (20-82)	50.4±14.4 (18-74)	54.8±15.1 (18-82)
**M: F**	69:111	34:36	21:29	124:176	4:38	19:34	23:72

*, Mean ± Standard deviation (Range)

Supplementary Figures

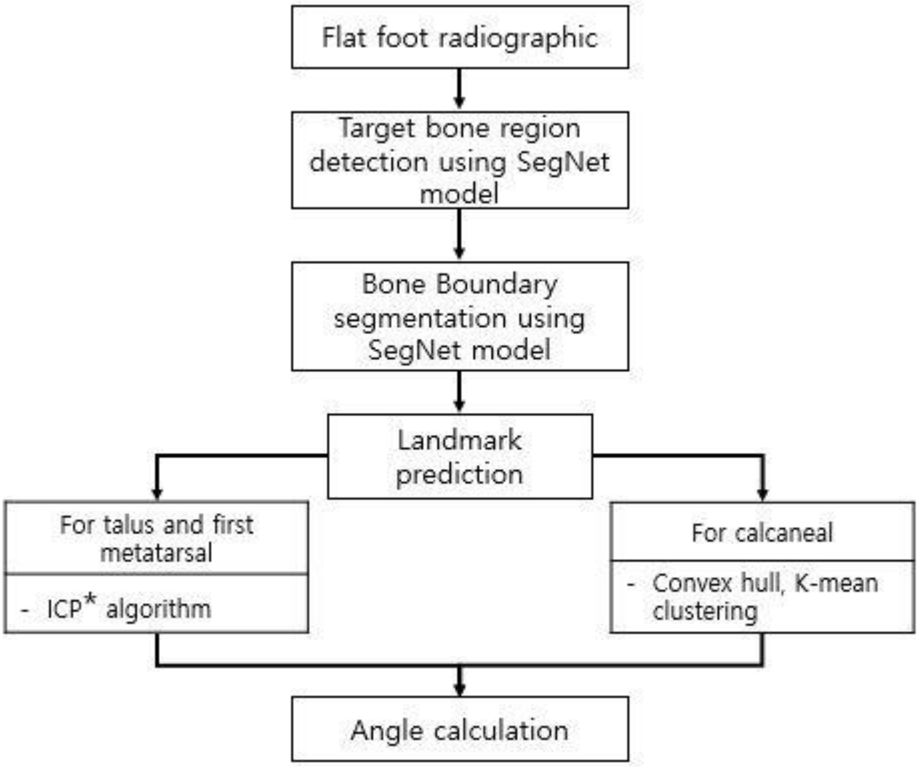

Supplementary Figure S1 Flowchart of the automatic angle measurement algorithm.

*, Iterative closest point

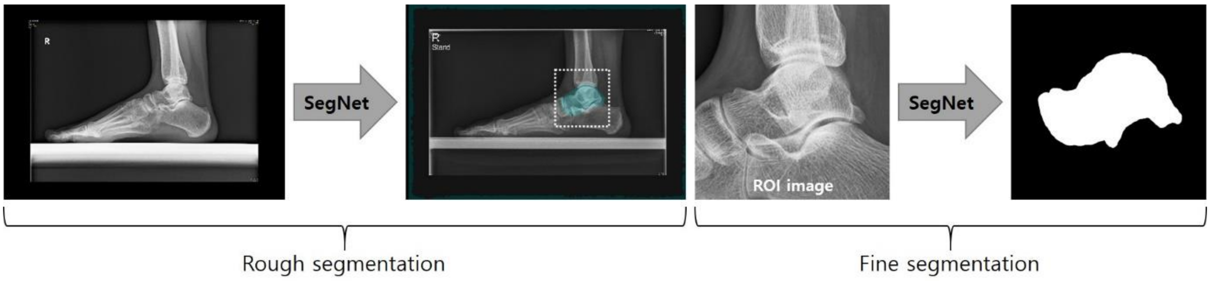

Supplementary Figure S2 Segmentation method using SegNet model.

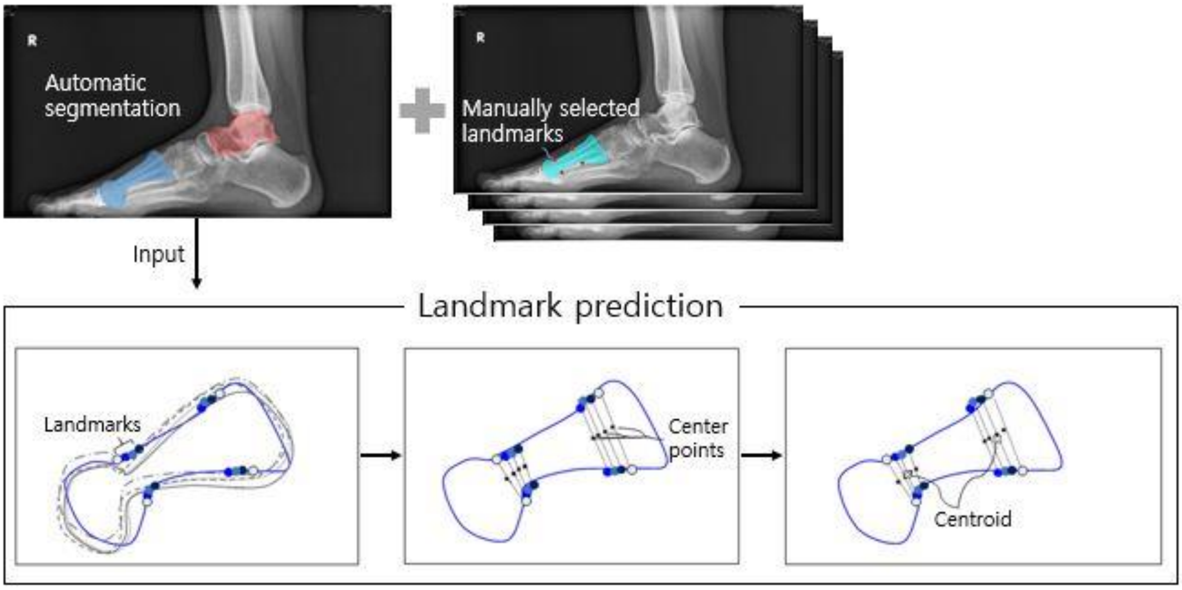

Supplementary Figure S3 Landmark prediction of talus and first metatarsus using ICP method.

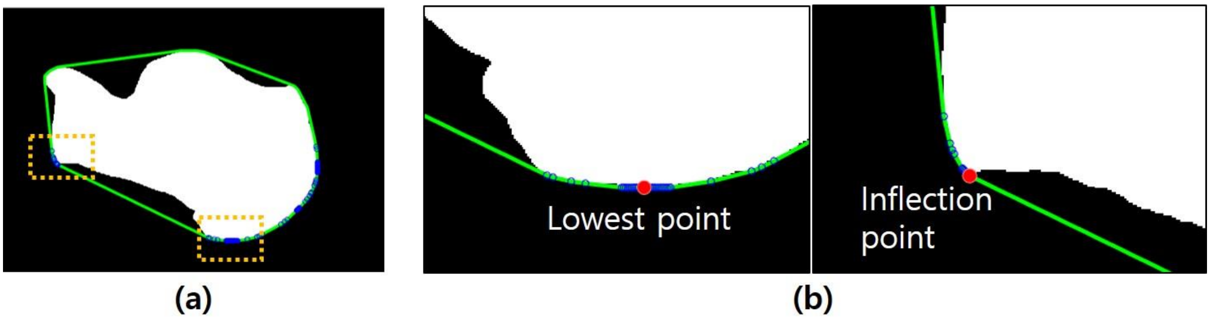

Supplementary Figure S4 Landmark prediction of calcaneus. (a) Extract landmark candidates from convex set. (b) The lowest point and the inflection point among the extracted landmark candidates.

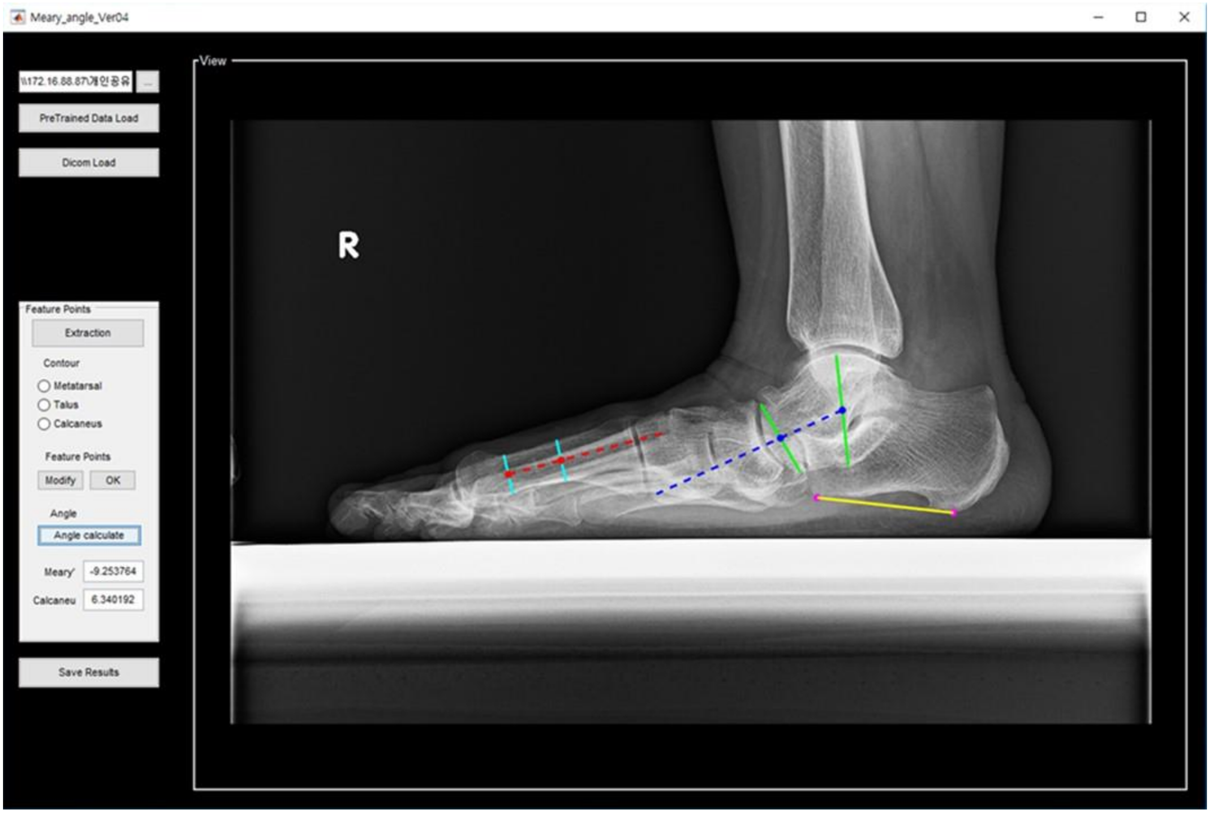

Supplementary Figure S5 How to use the with-algorithm tool. Before starting the program, specify the folder to save the results. Load Pre-trained-data. In the case of images without existing results, feature points are extracted using pre-trained data. If there is a result, the previously saved value is loaded. Load Dicom image to measure angle. Simple patient information is displayed for the imported image. Click the Extraction button to automatically generate feature points for measuring angles. If you want to check the outline of each bone, turn the Contour radio button on. Modify button can change the location of the feature point to be changed. Click the Angle calculate button to check the calculated Meary and calcaneus angles after checking the result, click the save button.

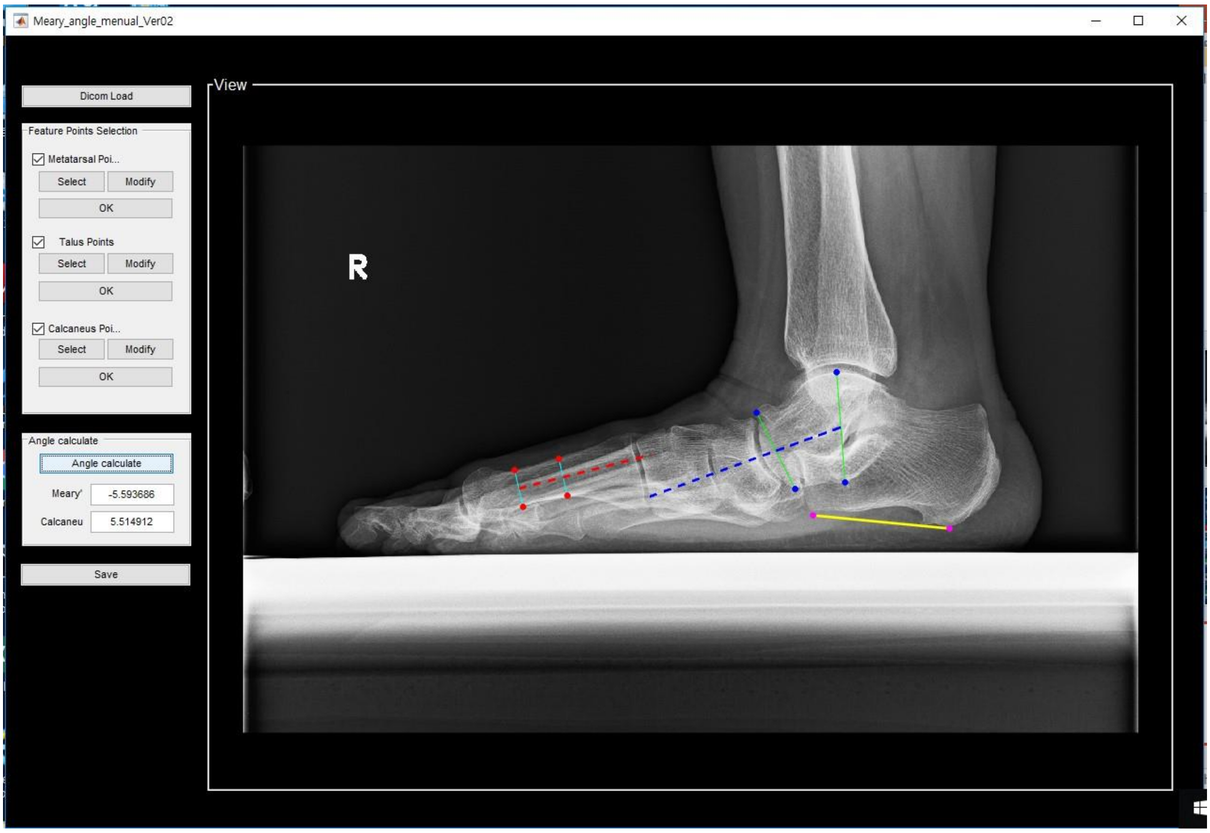

Supplementary Figure S6 How to use the without-algorithm tool. To manually select the feature points of each bone, click the "Select" button and specify the point. When the selection is complete, press the "OK" button. If you want to modify it, click the "Modify" button, then correct it and click the "OK" button Press. After all selections are made, click the "Angle calculate" button to automatically calculate the angle. Click "Save" and the result will be saved in the folder where the launcher is located.
